# Supplementary material for: Effects of Oxidative Stress on the Autophagy and Apoptosis of Granulosa Cells in Broody Geese
Source: Int J Mol Sci. 2023 Jan 21;24(3):2154. doi: 10.3390/ijms24032154 (PMC9916681; doi:10.3390/ijms24032154)
Supplement: Supplementary file 1 [file ijms-24-02154-s001.zip › ijms-2108870-supplementary.pdf]

Supplementary Materials:

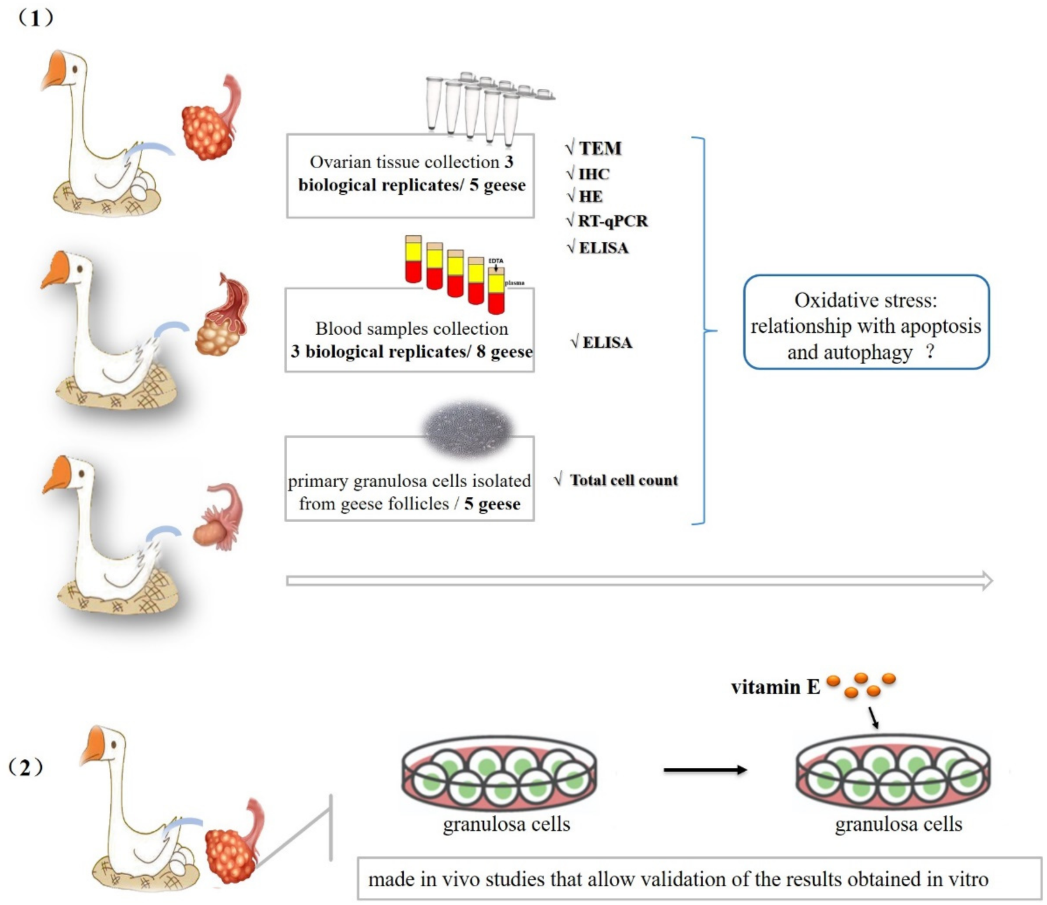

Figure S1. Experimental design and workflow for assessing the effects of oxidative stress on the autophagy and apoptosis of GCs in broody geese.
